# Temperature-triggered reversible breakdown of polymer-stabilized olive–silicone oil Janus emulsions†

**DOI:** 10.1039/c9ra03463c

**Published:** 2019-06-19

**Authors:** Rajarshi Roy Raju, Ferenc Liebig, Andreas Hess, Helmut Schlaad, Joachim Koetz

**Affiliations:** Institute of Chemistry, University of Potsdam Karl-Liebknecht-Str. 24-25 14476 Potsdam Germany koetz@uni-potsdam.de

## Abstract

A one-step moderate energy vibrational emulsification method was successfully employed to produce thermo-responsive olive/silicone-based Janus emulsions stabilized by poly(*N*,*N*-diethylacrylamide) carrying 0.7 mol% oleoyl side chains. Completely engulfed emulsion droplets remained stable at room temperature and could be destabilized on demand upon heating to the transition temperature of the polymeric stabilizer. Time-dependent light micrographs demonstrate the temperature-induced breakdown of the Janus droplets, which opens new aspects of application, for instance in biocatalysis.

## Abstract 2

A one-step moderate energy vibrational emulsification method was successfully employed to produce thermo-responsive olive/silicone-based Janus emulsions stabilized by poly(*N*,*N*-diethylacrylamide) carrying 0.7 mol% oleoyl side chains.

## Graphical abstract

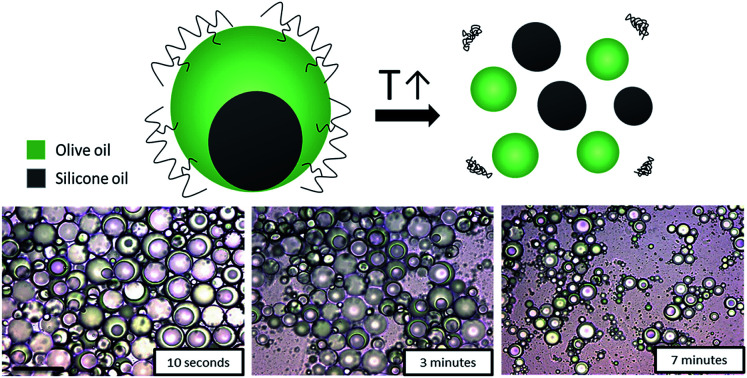

## 1. Introduction

Janus emulsions, which are composed of two immiscible oil components dispersed in a continuous aqueous medium, have attracted considerable interest due to their special properties, which are of relevance in different fields of application, for instance as drug delivery systems, and for preparing porous materials or anisotropic particles.^1,2^ The formation process can be realized under mild microfluidic conditions^3^ or by mixing in a minishaker.^2^ Friberg *et al.* recommended that the formation of Janus emulsions strongly depends on the interfacial tension (*γ*) between the different liquid phases.^4^ Numerical analysis has shown that the interfacial tension between the two oil phases must be in the order of 1 mN m^−1^ and <5 mN m^−1^ between the outer oil phase and water.^4,5^ Equilibrium calculations underline a direct correlation between the interfacial tension and the angles at the contact line between the three liquids in equilibrium, directing the type of Janus emulsion being formed.^5^ Therefore, a transition from a core–shell structure to a dumbbell or perfect Janus emulsion structure is driven by the interfacial tension. Further investigations evidenced that the configuration of the complex emulsion droplets can be tuned dynamically by exploiting interfacial tensions among the components.^6,7^ To adjust the interfacial tension at the oil/water interface surface active components are required. For making Janus emulsions of olive oil and silicon oil in water, the nonionic surfactant Tween®80 (polyethylene glycol sorbitan monooleate) can take over the role of the surface-active component very well, but also phospholipids can significantly decrease the surface tension between the three liquids, and therefore, stabilize Janus droplets of significant smaller dimensions.^8^ Recently, it was shown that surface-active polymers, *e.g.*, gelatin and chitosan, can also induce the formation of completely engulfed Janus droplets.^2^ By using particles, *i.e.*, soft gelatin/carboxymethylcellulose complexes^9^ or hard magnetite nanoparticles,^10^ one can obtain so-called Pickering Janus emulsions.

The use of stimuli-responsive polymeric emulsifiers for stabilizing Janus emulsions is of interest because this would offer the possibility to obtain “smart” Janus emulsions with special temperature-dependent properties. To the best of our knowledge, the instantaneous breakdown of Janus droplets by exploiting thermo-responsive copolymers has not been realized yet. However, it has been shown that poly(*N*-isopropylacrylamide) (PNIPAM)-based “smart” microgels can produce emulsions with very interesting temperature-dependent behavior.^11–15^ The internal structure of the soft gel particles at the oil–water interface is of high importance for the emulsification process.^16,17^ Furthermore, the adsorption behavior of the microgel particles in combination to the enzyme affinity is of special relevance for the application in biocatalysis.^18–20^

Another well-known thermo-responsive polymer is poly(*N*,*N*-diethylacrylamide) (PDEAA).^21^ However, the PDEAA homopolymer is not able to produce Janus emulsions. Therefore, we have synthesized thermo-responsive copolymers of *N*,*N*-diethylacrylamide (DEAA) and 2-oleoylethyl methacrylate (OEMA) with <1 mol% hydrophobic oleoyl side chains to stabilize Janus emulsions containing olive oil, silicone oil and water in comparison to our earlier studies.^8–10^ The resulting completely engulfed Janus emulsion droplets spontaneously disappeared upon heating the system to 35 °C. The Janus emulsions were examined by cryogenic scanning electron microscopy (cryo-SEM) and light microscopy.

## 2. Experimental

### 2.1 Materials

Olive oil, silicone oil with low viscosity (10 mPa s) and the hydrophobic dye Nile red were obtained from Sigma-Aldrich and used without further purification. Oleic acid (99%) was purchased from Fluka. *N*,*N*′-Dicyclohexylcarbodiimide (DCC, 99%) and 4-(dimethylamino)pyridine (DMAP, 99%) were obtained from Acros Organics. 2-Hydroxyethyl methacrylate (HEMA, 97%) and *N*,*N*′-diethylacrylamide (DEAA, 98%) were purchased from Alfa Aesarand TCI, respectively. 2,2′-Azobisisobutyronitrile (AIBN, 98%) from Sigma-Aldrich was recrystallized from isopropanol before use. Dichloromethane (DCM), anisole, and petroleum ether were received from Fisher Scientific.

2-Oleoylethyl methacrylate (OEMA) was synthesized by Steglich esterification of HEMA with oleic acid (see ESI†).^22^^1^H NMR (300 MHz, CDCl_3_): *δ* 6.11, 5.58 (d, 2H, C=C*H*_2_), 5.33 (m, 2H, –C*H*=C*H*–), 4.33 (m, 4H, –O–C*H*_2_–C*H*_2_–O–), 2.32 (t, 2H, O=C–C*H*_2_), 1.94 (m, 7H, CH_2_=C–C*H*_3_, –C*H*_2_–CH=CH–C*H*_2_–), 1.59 (m, 2H, O=CCH_2_–C*H*_2_–), 1.27 (m, 20H, –(C*H*_2_)_6_–CH_2_–CH=CH–CH_2_–(C*H*_2_)_4_–), 0.87 (m, 3H, –CH_2_–C*H*_3_) (Fig. S1†).

**Scheme 1:**
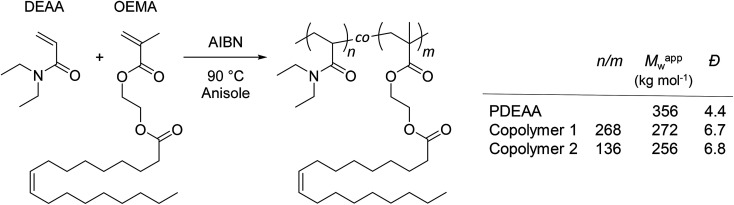
Synthesis and molecular characteristics of PDEAA and DEAA/OEMA copolymers 1 and 2. The relative molar ratios of DEAA/OEMA units (*n*/*m*) in the copolymers were determined by ^1^H NMR spectroscopy (300 MHz, CDCl_3_) from the peak integrals at 2.85–3.85 ppm (DEAA, –N–C*H*_2_–CH_3_) and 5.20–5.50 ppm (OEMA, –C*H*=C*H*–) (Fig. S2–S4†). Apparent weight-average molar masses (*M*^app^_w_) and dispersity indexes (*Đ*) were determined by size exclusion chromatography (SEC) using polystyrene calibration (Fig. S5†).

A homopolymer of DEAA (PDEAA) and two copolymers (copolymers 1 and 2) with different ratios of DEAA and OEMA were synthesized by free radical polymerization (see ESI† and Scheme 1).

### 2.2 Janus emulsion preparation

Aqueous solutions of DEAA/OEMA copolymer 2 (varying in the polymer concentration between 0.01 and 3 wt%) were prepared by suspending the polymer in water (pH 7–8) and stirring overnight. Afterwards, one-step vibrational emulsification was performed in a 10 mL glass tube by mixing olive oil, silicone oil and the aqueous emulsifier solution in a Minishaker IKA (Roth) at 2500 rpm for two minutes. Each mixture contained 70 vol% of the aqueous emulsifier solution, 15 vol% silicone oil, and 15 vol% olive oil.

### 2.3 Analytical instrumentation and methods

#### NMR spectroscopy

Proton nuclear magnetic resonance (^1^H NMR) spectra were recorded on Bruker Avance 300 MHz spectrometer at room temperature using CDCl_3_ as the solvent. Signals were referenced to the solvent peak at *δ* 7.26 ppm.

#### Size exclusion chromatography

Size exclusion chromatography (SEC) with simultaneous UV and RI (differential refractive index) detection was performed with THF as the eluent (flow rate of 0.5 mL min^−1^) at room temperature. The stationary phase was a 300 × 8 mm^2^ PSS SDV linear M column (3 μm particle size, molar mass range 10^2^–10^6^ Da). Solutions containing ∼0.15 wt% polymer were filtered through 0.45 μm PTFE filters; the injected volume was 100 μL. Polystyrene standards (PSS, Mainz, Germany) were used for calibration.

#### Turbidimetry

Turbidimetric measurements were conducted with a turbidimetric photometer TP1 (Tepper Analytik, Wiesbaden, Germany) operating at *λ* = 670 nm. Samples were and analyzed between 25 to 45 °C at a heating rate of 1.0 K min^−1^. The temperature at which the transmission dropped to 80% was taken as the cloud point temperature.

#### Interfacial tension measurements

Interfacial tension (*γ*) measurements were carried out with a Krüss digital tensiometer K10TS. The aqueous phase was taken into a glass pot and the cleaned platinum ring was placed on the surface. Then the upper oil phase was placed on the top, and the sample was allowed to stay for 5 minutes, before the ring was lifted up.

#### Light microscopy

Micrographs of the prepared emulsions were acquired by a Leica DMLB microscope, equipped with the Leica DFC 295 live camera. Images were captured at different magnifications from an emulsion drop placed over a glass slide (76 × 26 mm^2^) according to the procedure described earlier.^23^ For determination of the droplet size distribution of emulsions, the iTEM software was used to count the diameter of at least 500 individual drops.

Light microscopic observation of the temperature-dependent response of the emulsion droplets was accomplished by adapting LINKAM-PE-60 stage controller slides. The temperature of the emulsion droplet contained microscope slides was thermoelectrically controlled, while simultaneous imaging of the emulsion droplets was performed by the light microscope.

#### Dynamic light scattering

Dynamic light scattering (DLS) measurements were conducted with a Zetasizer Nano ZS from Malvern. The hydrodynamic diameter (*d*_H_) of the polymer chains or aggregates in dependence on the temperature was calculated by using the multimodal peak analysis of the intensity plot.

#### Cryo-scanning electron microscopy

To obtain structural information about copolymer as well as the individual emulsion droplets, cryo-scanning electron microscopy (cryo-SEM) was carried out by using Hitachi S-4800. In this procedure, samples were cooled down through nitrogen slush at atmospheric pressure, followed by freeze fracturing at −180 °C and etching 60 s at −98 °C. Afterwards, a thin platinum layer was used to sputter the samplesat the GATAN Alto 2500 cryo-preparation chamber. Finally, the samples were transferred to the cryo-SEM.

## 3. Results and discussion

PDEAA is a thermo-responsive polymer showing lower critical solution (LCST) behavior in aqueous solution. The turbidimetric analysis of a 2 wt% aqueous solution of PDEAA reveals a cloud point temperature of 30 °C (see Fig. S6†). The DEAA/OEMA copolymers 1 and 2 (Scheme 1), which can be regarded as hydrophobically modified PDEAA, exhibit virtually the same cloud point temperature as the PDEAA homopolymer. Hence, the incorporation of <1 mol% oleolyl chains into PDEAA has no detectable impact on the LCST behavior.

**Fig. 1:**
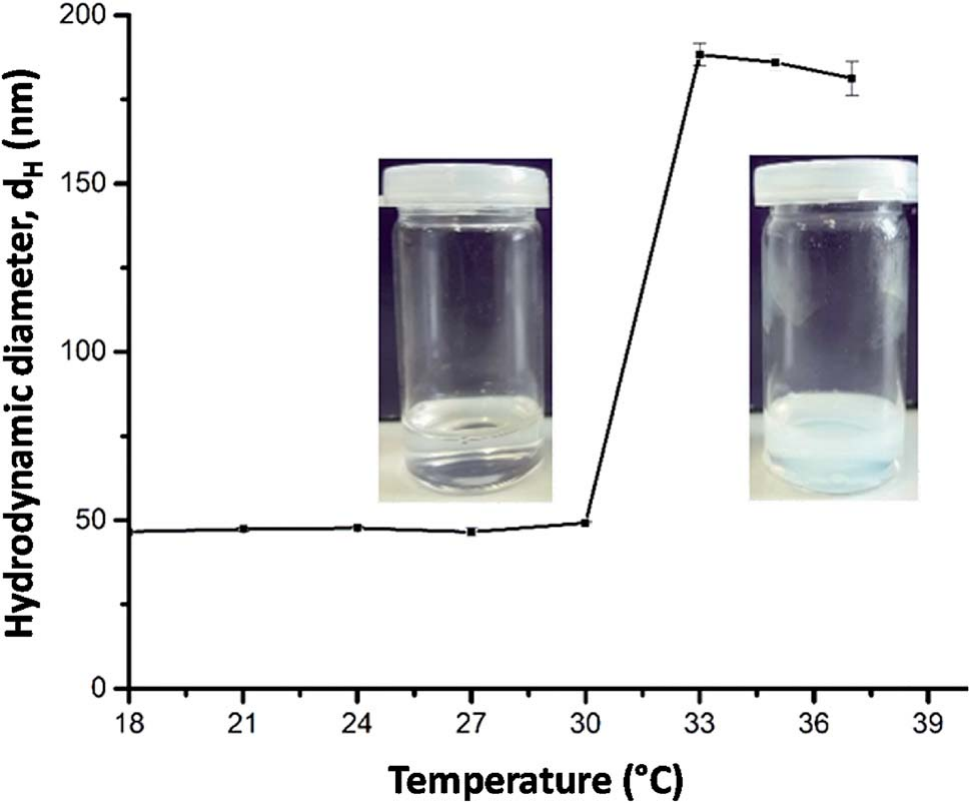
Temperature-dependent DLS data (hydrodynamic diameter *d*_H_) of a 2 wt% aqueous solution of copolymer 2 and corresponding photographs taken at below and above 32 °C.

First experiments showed that the PDEAA homopolymer did not fulfill the requirements to produce Janus emulsions. However, the DEAA/OEMA copolymers 1 and 2 were able to form Janus droplets between olive oil and silicone oil. With copolymer 1, however, phase separation was observed already after one hour, while copolymer 2, containing ∼0.7 mol% of hydrophobic oleoyl side chains, could stabilize Janus emulsions for more than three months. Addition of copolymer 2 to the aqueous solution leads to a decrease of the surface tension to (41.3 ± 0.7) mN m^−1^, and the interfacial tension at the olive oil/water interface decreases to (3.5 ± 0.5) mN m^−1^. Therefore, the requirements to form a Janus emulsion (<4–5 mN m^−1^) are fulfilled,^5^ and significant smaller droplets are formed with increasing polymer concentration. Additionally performed DLS experiments of a 2 wt% aqueous polymer solution at room temperature indicates the presence of single polymer chains or small aggregates with a hydrodynamic diameter (*d*_H_) of about 50 nm (Fig. 1). Temperature dependent DLS data show a strong change of *d*_H_ between 30 and 32 °C (Fig. 1). The spontaneous increase of the diameter at 32 °C up to 200 nm indicates the aggregation process due to the temperature-induced collapse of the polymer at the volume phase transition temperature (VPTT), a typical feature of thermo-responsive polymers.^19,21^

**Fig. 2:**
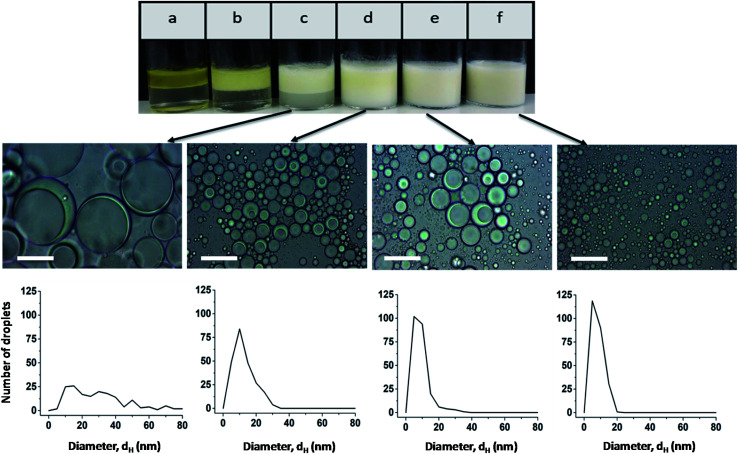
Photographs and light micrographs (scale bar = 50 μm) with the corresponding histograms of freshly prepared emulsions at different copolymer concentrations: (a) 0.01 wt%, (b) 0.1 wt%, (c) 0.5 wt%, (d) 1 wt%, (e) 2 wt%, and (f) 3 wt%.

The effect of the stabilizer concentration on the stability of the emulsion was examined one hour after preparation up to a polymer concentration of 3 wt% (Fig. 2). Higher polymer concentrations are not useful due to the strong viscosifying effect. The corresponding micrographs of emulsions a and b are shown in Fig. S7.† At the lowest stabilizer concentration of 0.01 wt%, a phase separation between oil and water occurs (sample a). Sample b and c also show a phase separation. The upper turbid phase of sample b contains Janus emulsion droplets, as can be seen by the corresponding light micrograph in Fig. S7b.† At stabilizer concentrations >0.5 wt%, no phase separation was observed for the freshly prepared emulsions, and the corresponding light micrographs confirm the formation of double emulsion droplets. Most of the droplets show the typical topology of completely engulfed Janus droplets, in which the silicone oil core is covered by an olive oil shell. Evidently, a minimum amount of stabilizer is required to form Janus emulsion droplets. Furthermore, the size of the Janus droplets is significantly affected by the stabilizer concentration. Larger droplets with diameters of about 80 μm are formed at lower copolymer concentrations of ≤0.5 wt%, and the droplet size decreases significantly with increasing copolymer concentration. The size distribution becomes more distinct at ≥1 wt% copolymer concentration (samples d, e and f) with a main droplet fraction of about 10 μm, as can be seen by the corresponding histograms in Fig. 2.

**Fig. 3:**
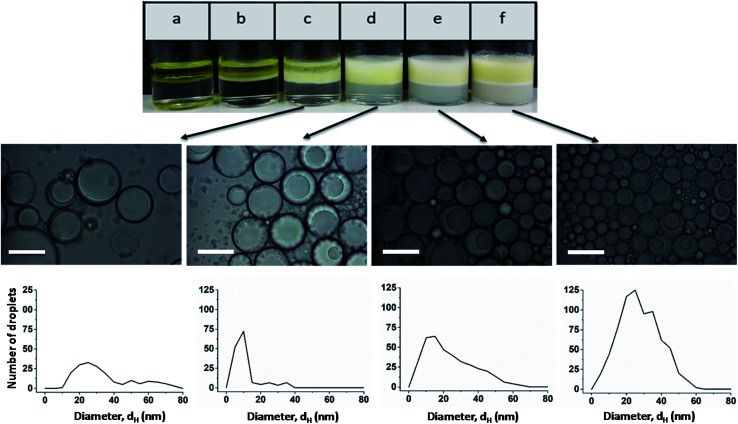
Photographs and light micrographs with the corresponding histogram of emulsions at different copolymer concentrations after one month: (a) 0.01 wt%, (b) 0.1 wt%, (c) 0.5 wt%, (d) 1 wt%, (e) 2 wt%, and (f) 3 wt%.

Light micrographs taken after one month show that stable Janus emulsion droplets of different size are formed only at a polymer concentration of ≥0.5 wt% (Fig. 3; the micrographs of emulsions a and b are shown in Fig. S8†). However, there is a time-dependent shift of the droplet size to larger dimensions (compare histograms in Fig. 2 and 3), which can be attributed to Ostwald ripening. At low copolymer concentration (sample c), a very broad size distribution is observed, which becomes more distinct at 1 wt% (sample d) with a mean droplet size of 10 μm, similar to the freshly prepared samples shown in Fig. 2. At higher copolymer concentrations, the main fraction is shifted from 10 μm to 25 μm accompanied by a peak-broadening at 3 wt% (sample f). Furthermore, the photographs taken after one month show a separation into two phases with different turbidity (compare Fig. 3). A more detailed investigation of the bottom phase and the more turbid top phase leads to the conclusion that larger Janus droplets are in the upper phase. This phenomenon can be related to a density profile of Janus droplets of different size. Taking into account that the density of olive oil (0.909 g cm^−3^) is lower than that of silicon oil (0.934 g cm^−3^),^2^ one can assume that completely engulfed larger droplets moved into the upper phase.

**Fig. 4:**
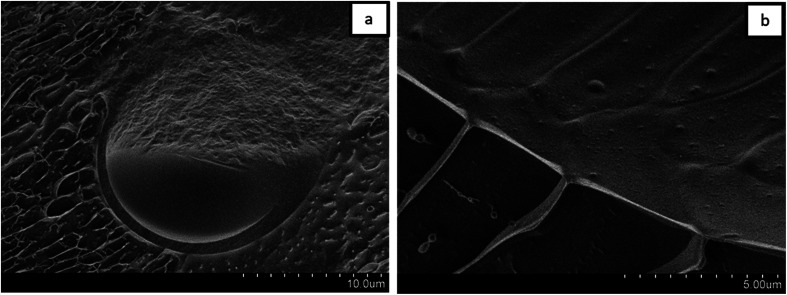
Cryo-SEM micrographs of a Janus droplet stabilized by a copolymer layer.

**Scheme 2:**
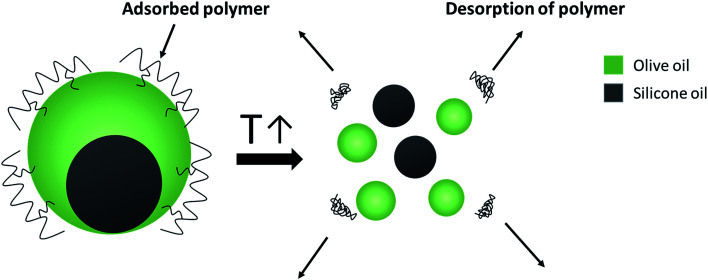
Spontaneous breakdown of Janus emulsions triggered by the temperature-induced copolymer collapse.

For a better understanding of the stabilization mechanism of the Janus droplets, we have performed cryo-SEM experiments. The micrograph in Fig. 4a shows a large, completely engulfed Janus droplet. At higher magnification (Fig. 4b) one can see that the olive oil shell is covered by a thin adsorption layer. Additionally performed cryo-SEM investigation on the polymer solution shows a similar picture of segregation, which lead us to the conclusion that the olive oil shell is stabilized by a layer of the copolymer. To adsorb DEAA/OEMA copolymers at the olive oil interface we need a certain number of OEMA monomer units with hydrophobic side chains, which are able to diffuse into the olive oil (represented in Scheme 2). Evidently, already the ∼0.7 mol% oleolyl side chains in copolymer 2 are sufficient to achieve this task.

**Fig. 5:**
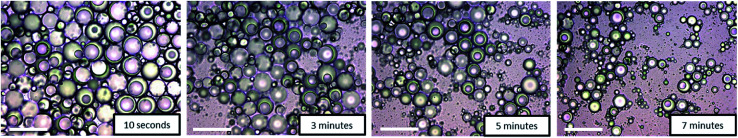
Time-dependent disappearance of Janus droplets by “immediate explosion” at 35 °C. Micrographs taken after 10 s, 3 min, 5 min, and 7 min (scale bar = 100 μm).

The temperature-responsiveness of the emulsion was investigated by heating the emulsion sample to above 32 °C (see Fig. 1). An optical inspection already shows a flocculation of the whole system at a temperature of >32 °C. For a more detailed characterization, the sample was prepared at room temperature and temperature-dependent evolution was examined with light microscopy after one day of preparation. A drop of emulsion was placed on the microscope slide and the sample was heated slowly from 20 °C to 35 °C. No significant change was observed until the temperature of the slide reached 35 °C, which is slightly above the VPTT of copolymer 2. At this temperature, individual Janus droplets spontaneously disappeared as can be seen in Fig. 5. After 7 minutes at 35 °C, most of the larger Janus droplets disappeared, and after 20 minutes no droplets could be observed. The “immediate explosion” of the droplets, which is demonstrated in a video (ESI†), can be related to the collapse of the polymeric adsorption layer at the droplet surface, followed by the desorption of the polymer.

This phenomenon is illustrated in the sketch in Scheme 2. After desorption of the copolymer layer, Janus droplets are no longer stable and disappear spontaneously. Similar effects were observed with PNIPAM-based microgel particles adsorbed at the oil/water interface below and above the VPTT.^24^ At *T* < VPTT, the swollen PNIPAM microgels can spread at the droplet surface and cover the interface, while at *T* > VPTT, the spreading ability is decreased due to a change of the interfacial tension. Therefore, a higher number of particles is needed to cover the interface.^24^ This is in agreement with our observation that the larger droplets disappear first and later the smaller ones (compare Fig. 5). Former experiments with chitosan in dependence on pH show similar effects: chitosan in the extended conformation at pH 2 stabilizes significantly larger Janus droplets as compared to when it is in a more entangled conformation at pH 5.^10^

**Fig. 6:**
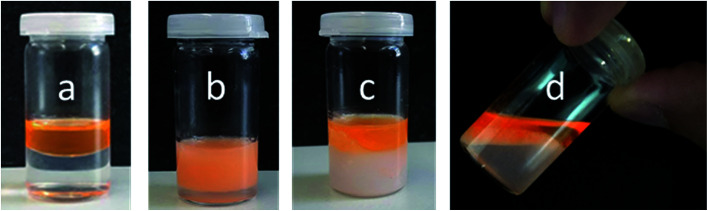
Temperature-triggered breakdown behavior of Janus emulsions. Olive oil and silicone oil were stained by Nile red dye (0.1 mM mL^−1^ oil): (a) before emulsification by copolymer 2, (b) after emulsification, (c) after heating the sample at 35 °C for 20 minutes, (d) after cooling down the sample to room temperature for 15 minutes.

Further investigations were conducted to underline the mechanism of the temperature-triggered breakdown of Janus emulsions. The olive oil and silicone oil were stained by lipophilic dye “Nile red” prior to emulsification by 1 wt% copolymer 2 solution at room temperature (Fig. 6a). After shaking the vial, an orange colored emulsion was obtained (Fig. 6b). Light microscopic investigations show the formation of completely engulfed Janus emulsion droplets as observed in previous experiments performed in absence of the dye, suggesting that the dye molecules did not significantly affect the emulsification process. A phase separation was not evident by visual inspection of the emulsified sample. Afterwards, the emulsion sample was heated to 35 °C and the temperature was kept constant for 20 minutes. The increase of temperature resulted in an initial flocculation of the sample followed by a complete phase separation into the oil and water phases (Fig. 6c), which was readily observed after 20 minutes and supported by the light microscopic image of the sample without Janus droplets. The turbid ‘milky white’ bottom phase of the destabilized sample represents the collapsed copolymer above its VPTT temperature and colored top phase denotes the dye-stained oil components. This observation clearly suggests the triggered breakdown of the Janus emulsion due to the collapse and desorption of the copolymer into the aqueous phase. After cooling the sample to room temperature and waiting for 15 minutes, the aqueous phase becomes more transparent again indicating a re-solubilization of the copolymer in water, which paves the way back to initial state before emulsification. Therefore, the whole process of emulsification and phase separation is reversible.

Samples can be re-emulsified after shaking at 2500 rpm. No significant changes in droplet size and shape were observed after re-emulsification. This ought to be of relevance for the recycling Janus emulsions after enzyme reaction according to the concept of smart emulsions for biocatalysis.^19^ The ability of re-emulsification clearly verifies the presence of Janus droplets in the equilibrium state at room temperature, in full agreement with equilibrium calculations of Janus droplets.^5^

## 4. Conclusions

Janus emulsions were prepared by using a thermo-responsive DEAA/OEMA copolymer with ∼0.7 mol% hydrophobic oleoyl side chains. The resulting partially engulfed emulsion droplets of silicone oil and olive oil were long-time stable at room temperature. Upon heating the Janus emulsions to 35 °C, a spontaneous “explosion” of the Janus droplets occurred, which can be related to the polymer collapse at the droplet interface accompanied by the polymer desorption. Accordingly, the Janus emulsion was destabilized resulting in phase separation. This behavior can be used for catalytic processes performed in the Janus emulsions, and the separation of the resulting components according to their hydrophobicity, exemplarily shown for the dye Nile red. The temperature-dependent reversibility of droplet stabilization and destabilization is a special feature of these thermo-responsive Janus emulsions and opens new aspects of application.

## Conflicts of interest

There are no conflicts to declare.

## Supplementary Material

RA-009-C9RA03463C-s001

RA-009-C9RA03463C-s002
