# Supplementary material for: Temperature-triggered reversible breakdown of polymer-stabilized olive–silicone oil Janus emulsions
Source: RSC Adv. 2019 Jun 19;9(34):19271–7. doi: 10.1039/c9ra03463c (PMC9064947; doi:10.1039/c9ra03463c)
Supplement: RA-009-C9RA03463C-s001 [file RA-009-C9RA03463C-s001.pdf]

Electronic Supplementary Material (ESI) for RSC Advances.
This journal is © The Royal Society of Chemistry 2019

Supporting Information

Temperature-triggered reversible breakdown of polymer-stabilized olive-silicone oil Janus emulsions

Rajarshi Roy Raju, Ferenc Liebig, Andreas Hess, Helmut Schlaad, Joachim Koetz*

Institute of Chemistry, University of Potsdam, Karl-Liebknecht-Str. 24-25, 14476 Potsdam, Germany

Synthesis of 2-oleoylethyl methacrylate (OEMA)

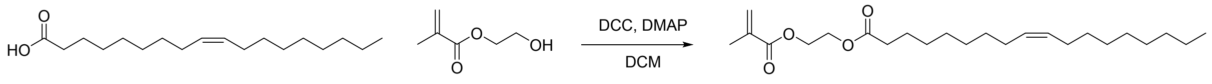

Oleic acid **HEMA** **OEMA**

2-Oleoylethyl methacrylate (OEMA) was synthesized according to Pipertzis et al. *ACS Macro Lett.*, 2018, **7**, 11–15. Oleic acid (5.00 g, 17.7 mmol) and DMAP (0.13 g, 1.2 mmol) were dissolved in DCM (20 mL) and cooled to 0 °C. Then, a solution of DCC (4.11 g, 20.0 mmol) in 5 mL of DCM was added to the reaction mixture, followed by the addition of HEMA (2.60 g, 20.0 mmol). The mixture was stirred at 0 °C for 20 min and at room temperature for 24 h. The reaction mixture was filtered and the filtrate washed with deionized water (45 mL) and four times with sodium bicarbonate solution. The solvent was removed under reduced pressure and the residue was purified by column chromatography (silica gel, hexane/ethyl acetate 95:5 v/v, R_f 0.22) to receive the OEMA as a colorless liquid; yield 80%.

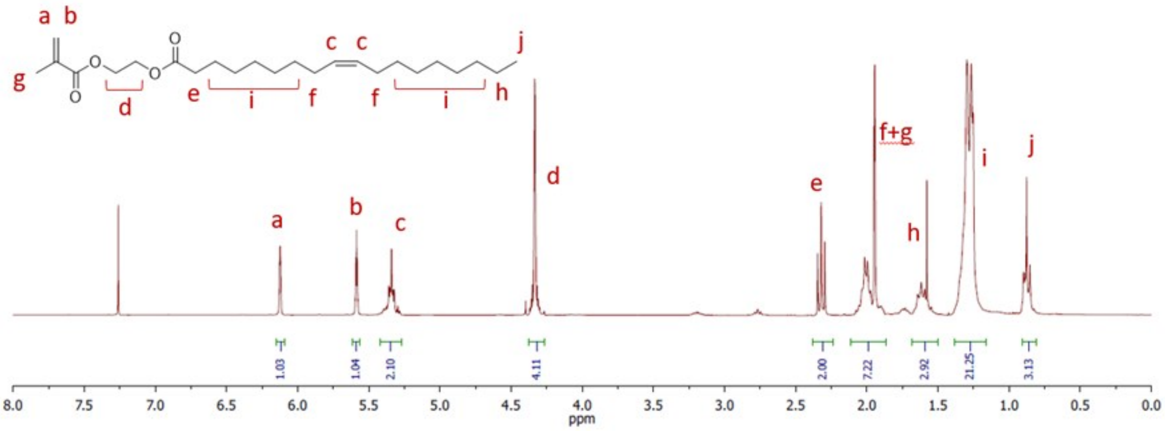

Figure S1. ^{1}HNMR spectrum (300 MHz) of OEMA in $CDCl_3$.

Polymerization

A homopolymer of DEAA (PDEAA) and two copolymers (copolymers 1 and 2) with different ratios of OEMA and DEAA were synthesized by free radical polymerization. Exemplarily, DEAA (3.00 g, 23.589 mmol), OEMA (0.031 g, 0.079 mmol), 0.10 mL of AIBN stock solution (13 mg, 0.079 mmol, in 1.0 mL anisole), and 1.5 mL of anisole were placed in a 8 mL-vial equipped with a septum. The solution was degassed via three freeze-pump-thaw cycles. Then, the mixture was stirred at 90 °C for 1 h, and the polymerization was quenched by cooling the reaction mixture in an ice-bath. The product was precipitated in petroleum ether, dissolved in DCM and precipitated three times into petroleum ether.

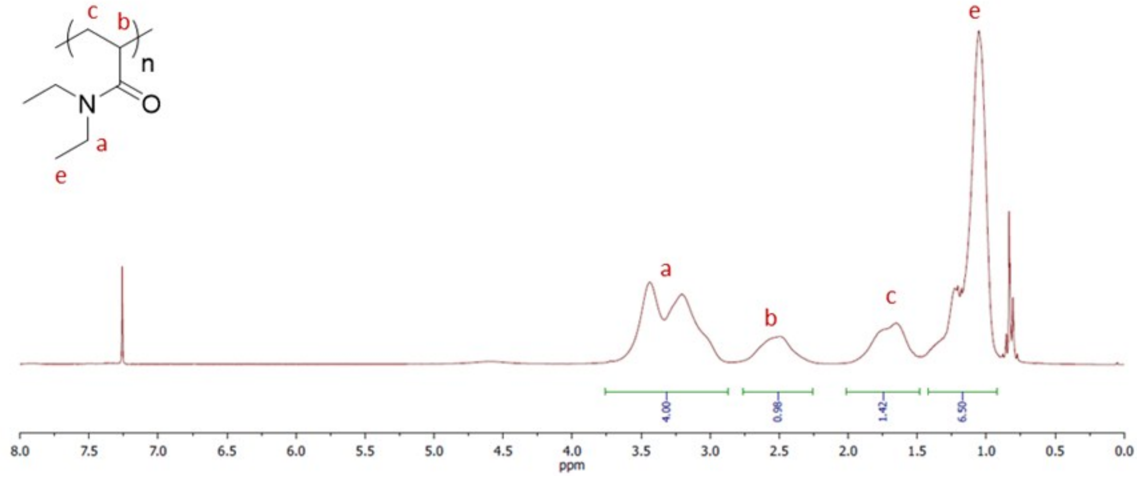

Figure S2. ^{1}HNMR spectrum (300 MHz) of homopolymer PDEAA in $CDCl_3$.

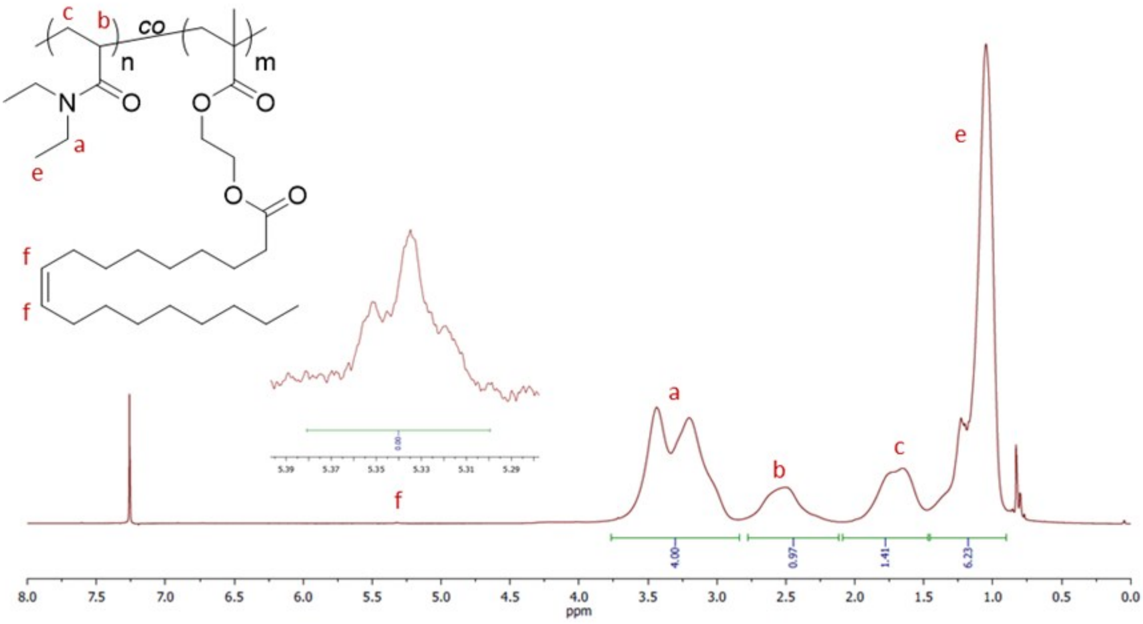

Figure S3. ^{1}H NMR spectrum (300 MHz) of copolymer 1 in $CDCl_3$.

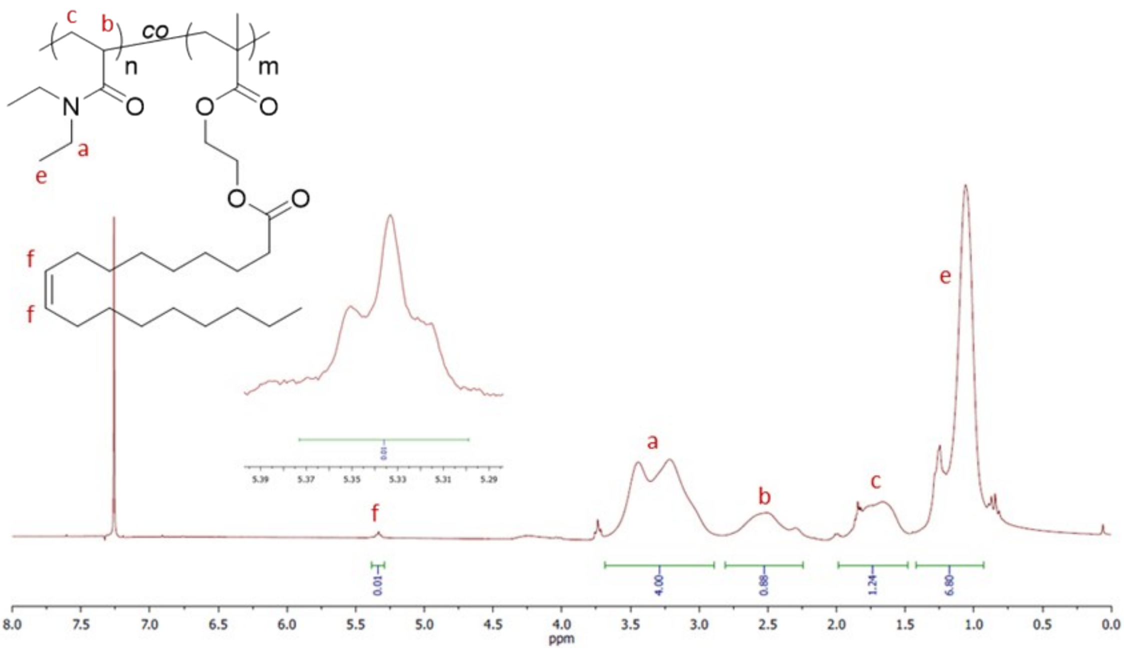

Figure S4. ^{1}H NMR spectrum (300 MHz) of copolymer 2 in $CDCl_3$.

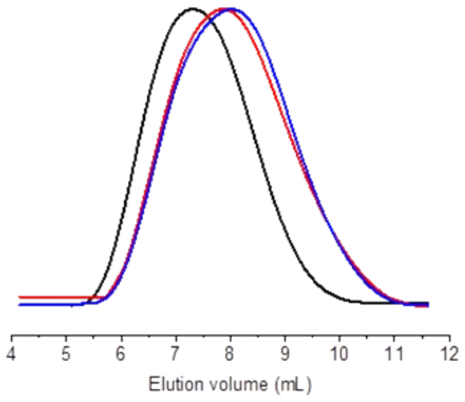

Figure S5. SEC-RI traces (eluent: THF) of homopolymer PDEAA (black), copolymer 1 (blue), and copolymer 2 (red).

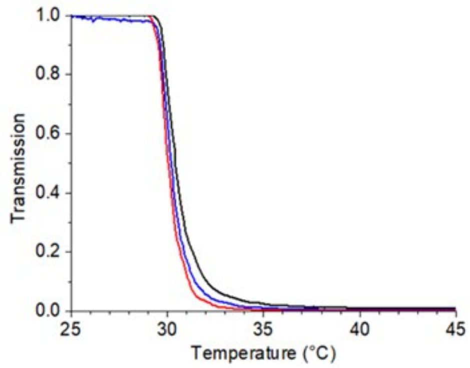

Figure S6. Turbidimetric heating curves (1 $Kmin^{-1}$) for 2 wt% aqueous solutions of PDEAA (black), copolymer 1 (blue), and copolymer 2 (red).

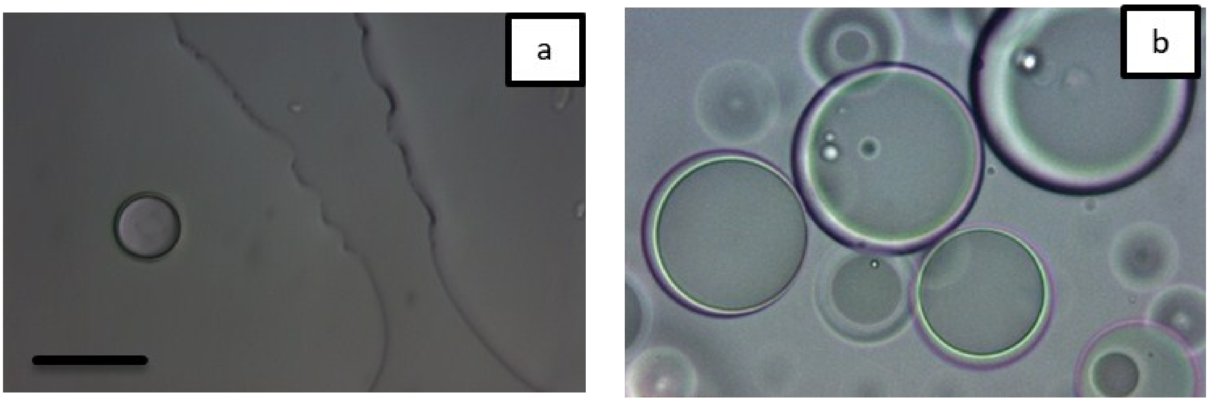

Figure S7. Light micrographs (scale bar = 50 μm) of freshly prepared emulsions at a copolymer concentrations of (a) 0.01 wt% and (b) 0.1 wt%.

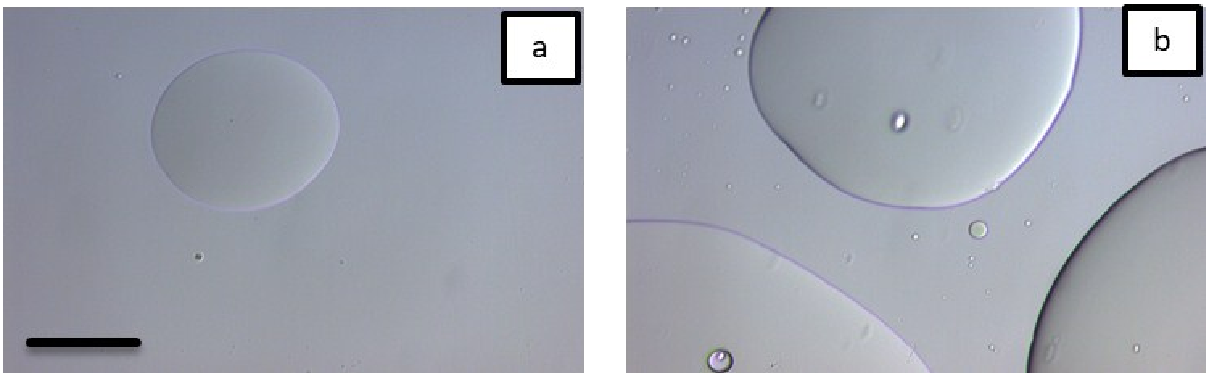

Figure S8. Light micrographs (scale bar = 50 μm) of emulsions after one month at a copolymer concentrations of (a) 0.01 wt% and (b) 0.1 wt%.
